# Comparison of total parathyroidectomy without autotransplantation and without thymectomy versus total parathyroidectomy with autotransplantation and with thymectomy for secondary hyperparathyroidism: TOPAR PILOT-Trial

**DOI:** 10.1186/1745-6215-8-22

**Published:** 2007-09-18

**Authors:** Katja Schlosser, Johannes A Veit, Stefan Witte, Emilio Domínguez Fernández, Norbert Victor, Hans-Peter Knaebel, Christoph M Seiler, Matthias Rothmund

**Affiliations:** 1 Department of Visceral, Thoracic and Vascular Surgery, Philipps University, Marburg, Germany; 2 Study Center of the German Surgical Society (SDGC), University of Heidelberg, Germany; 3 Institute of Medical Biometrics and Informatics (IMBI), University of Heidelberg, Germany

## Abstract

**Background:**

Secondary hyperparathyroidism (sHPT) is common in patients with chronic renal failure. Despite the initiation of new therapeutic agents, several patients will require parathyroidectomy (PTX). Total PTX with autotransplantation of parathyroid tissue (TPTX+AT) and subtotal parathyroidectomy (SPTX) are currently considered as standard surgical procedures in the treatment of sHPT. Recurrencerates after TPTX+AT or SPTX are between 10% and 12% (median follow up: 36 months).

Recent retrospective studies demonstrated a lower rate of recurrent sHPT of 0–4% after PTX without autotransplantation and thymectomy (TPTX) with no higher morbidity when compared to the standard procedures. The observed superiority of TPTX is flawed due to different definitions of outcomes, varying follow up periods and different surgical treatment strategies (with and without thymectomy).

**Methods/Design:**

Patients with sHPT (intact parathyroid hormone > 10 times above the upper limit of normal) on long term dialysis (>12 months) will be randomized either to TPTX or TPTX+AT and followed for 36 months. Outcome parameters are recurrence rates of sHPT, frequencies of reoperations due to refractory hypoparathyroidism or recurrent/persistent hyperparathyroidism, postoperative morbidity and mortality and quality of life. 50 patients per group will be randomized in order to obtain relevant frequencies of outcome parameters that will form the basis for a large scale confirmatory multicentred randomized controlled trial.

**Discussion:**

sHPT is a disease with a high incidence in patients with chronic renal failure. Even a small difference in outcomes will be of clinical relevance. To assess sufficient data about the rate of recurrent sHPT after both methods, a multicentred, randomized controlled trial (MRCT) under standardized conditions is mandatory.

Due to the existing uncertainties the calculated number of patients necessary in each treatment arm (n > 4000) makes it impossible to perform this study as a confirmatory trial. Therefore estimates of different outcomes are performed using a pilot MRCT comparing 50 versus 50 randomized patients in order to establish a hypothesis that can be tested thereafter.

If TPTX proves to have a lower rate of recurrent sHPT, no relevant disadvantages and no higher morbidity than TPTX+AT, current surgical practice may be changed.

**Trial registration:**

International Standard Randomized Controlled Trial Number Registration (ISRCTN86202793)

## Background

### Medical problem

Secondary hyperparathyroidism (sHPT) is common in patients with chronic renal failure, affecting most of those who are receiving hemodialysis [1,2]. Overall 60.992 patients were treated with continuous haemodialysis for renal failure in Germany 2004. The annual increase of 3% in patients requiring haemodialysis appears constant within the last years [3].

Renal insufficiency leads to a reduced synthesis of 1,25-dihydroxy-Vitamin D secretion and a decrease in phosphate excretion resulting in hypocalcemia and hyperphospatemia. Consecutively, parathyroid hormone secretion and parathyroid cell hyperplasia increases. With time, sHPT is characterized by persistently elevated levels of intact parathyroid hormone (PTH) above the desired target range recommended by the Kidney Disease Outcomes Quality Initiative (KDOQI [4]) and complicated by important disturbances in mineral metabolism and skeletal resistance to the calcemic actions of parathyroid hormone [5-7]. Renal osteopathy is the most widely recognized consequence of sHPT.

However, several reports indicate, that alterations in calcium and phosphorus metabolism, as a result of either sHPT or the therapeutic measures used to manage it, contribute to soft-tissue and vascular calcification, cardiovascular disease, and the risk of death [8-12].

Episodes of hypercalcemia and hyperphosphatemia are often aggravated by the use of calcium as a phosphate-binding agent, particularly in combination with Vitamin D sterols, which increase the absorption of calcium and phosphorus [12-15]. Recently, several new agents including calcimimetics [16,17], new phosphate binders [18] and less calcemic vitamin D analogues [19-22], have been added to the list of therapeutic modalities in the management of sHPT.

Despite the advent of these new therapeutic agents, patients with long-standing sHPT often require parathyroidectomy. The indication for surgery depends on clinical symptoms of progressive renal osteopathy and impaired calcemic response following PTH secretion in the absence of Vitamin D deficiency: either if hypercalcemia develops (spontaneously or under treatment with Vitamin D), if the new therapeutic modalities fail to control sHPT or if medical therapy has to be adjourned because of adverse reactions. However, the rate of parathyroidectomies (PTX) did not change significantly between 1991 and 2001 despite the progression in medical therapy with phosphate binders and Vitamin D analogues [23].

Overall PTX is required in about 20% of patients after 3–10 years of dialysis and up to 40% after 20 years [24,25].

### Current treatment options

Three different surgical procedures are reported in the literature following the first PTX for secondary HPT in 1960 [26]: subtotal PTX (removal of three and a half glands and leaving half of a gland as a remnant in the neck [27,28], total PTX with autotransplantation (TPTX+AT) of some of the excised tissue into defined areas (forearm muscle, anterior tibial muscle or subcutaneously [28-31], and total parathyroidectomy without autotransplantation (TPTX) [32,33]. Ogg [34] initially reported that TPTX provides a feasible therapeutic option in patients who are on chronic dialysis therapy. However, TPTX was not introduced into clinical practice because of the potential complication of adynamic bone disease or severe ongoing hypocalcemia. Therefore subtotal PTX or TPTX+AT, both supplemented with thymectomy, are currently considered as the standard procedures in the treatment of sHPT [28,32,35,36].

However, both procedures leave a fragment of activated, proliferated parathyroid tissue, though at different sites. Since the pathophysiological condition of chronic renal failure and maintenance dialysis continues, the growth stimulus persists and may cause recurrent sHPT at the site of the remnant. Although relapse of HPT has been observed at different intervals after initial surgery, it is quite clear that the incidence of recurrent disease increases with time [37,38]. A wide range of recurrence rates has been published for both procedures (range 5–80%). This variation is dependent on the definition of recurrent sHPT and variability in follow up periods given in different series for both techniques [32]. Recurrence-rates after TPTX+AT or subtotal PTX after a median follow up of 36 months are reported to be between 10% and 12% [31,39-41]. All other clinically relevant outcome parameters (mortality and morbidity) do not differ significantly between TPTX+AT and subtotal PTX [28,40,42].

**Figure 1:**
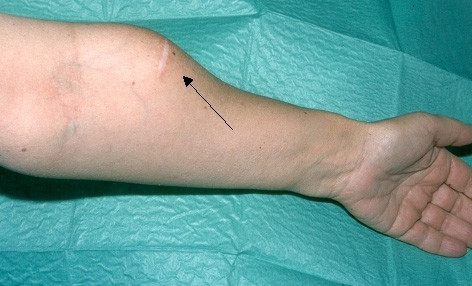
This figure shows the enlargement of a parathyroid autograft in a patient with recurrent secondary hyperparathyroidism, demonstrated by a knob underneath the scar.

The site of recurrence in patients after TPTX+AT is located in approximately 80% at the graft (Figure 1) and in 20% in the neck [24,36,43-47].

Graft-dependent recurrent sHPT after TPTX+AT can easily be cured by excision of the parathyroid autotransplant under local anaesthesia. If an operation is necessary for recurrent sHPT after subtotal PTX, reexploration of the neck is mandatory in all patients including the necessity of general anaesthesia and the increased risk of recurrent laryngeal nerve palsy [48]. Therefore, in case of recurrent disease, subtotal PTX as preceding procedure is clearly inferior to TPTX+AT given equal effectiveness for the initial treatment of sHPT.

Total parathyroidectomy without autotransplantation and without routinely performed thymectomy (TPTX) may provide an alternative strategy to the currently performed procedures mainly because of the reported lower rate of recurrent sHPT of 0–4% during long-term follow up [32,36]. Patients after TPTX did not develop hypoparathyroidism [32,36] and particularly adynamic bone disease as initially expected [32,36,49-52]. Moreover, patients lacking a parathyroid remnant maintained on a noncritical calcium/phosphate product during the ongoing follow-up, compared to individuals with persisting parathyroid secretion after PTX [38].

One explanation for the absence of serious adverse events may be that thymectomy was not performed on a regular basis. The inferior parathyroid glands and the thymus derive together from the third pharyngeal pouch. Even in case of resection of all four parathyroid glands (TPTX) residual microscopic parathyroid tissue or supernumerary glands may be located in the thymus preventing the patients to develop hypoparathyroidism and its sequelae [29,40,53-56].

### Aim of the study

The objective of this trial is to compare the different outcomes of two different surgical strategies (TPTX+AT+ versus TPTX) for secondary hyperparathyroidism over a three-year follow-up period.

Reducing the risk for recurrent sHPT is an important factor for the patients and the health care system, as surgery for recurrent sHPT is associated with high costs for localization, a lesser cure-rate and a higher morbidity when compared to intial parathyroidectomy [57]. All attempt should be done to reduce the risk of its development.

Further more, TPTX without parathyroid autotransplantation and without thymectomy is associated with a reduced operating time when compared to TPTX+AT thus leading to reduced costs when compared to TPTX+AT.

### Need for this study

To date, no prospective, randomized study exists comparing TPTX to the standard surgical procedures. The existing evidence of the potential superiority of TPTX regarding the recurrence rate is flawed due to mainly methodological problems of studies with different definitions of outcomes, varying follow up periods, different surgical treatment strategies (with and without thymectomy) and their retrospective design. Therefore bias and chance are not sufficiently controlled and the results of the present studies have to be interpreted with caution.

The difference observed between the rates of recurrent sHPT of TPTX+AT and TPTX seems to be of clinical relevance. To assess the true rates of both methods, a multicentred, randomized controlled trial (MRCT) under standardized conditions is mandatory to achieve high internal as well as external validity.

Due to the existing uncertainties the calculated number of patients necessary in each treatment arm (n > 4000) makes it impossible to perform this study as a confirmatory trial.

Therefore estimates of different outcomes are performed using a pilot MRCT comparing 50 versus 50 randomized patients in order to establish a hypothesis that can be tested thereafter. This seems to be feasible and ethical.

## Methods/Design

### Objectives of the study

The objective of this study is to compare different outcomes of TPTX and TPTX+AT for sHPT over a three-year follow-up period. The information gained will be used for further clinical trials.

The primary objective of this trial is to assess a better estimate of the rates of recurrence of both methods. This shall establish a hypothesis that can be tested thereafter.

Secondary objectives are divided into those occurring intraoperative, postoperative until discharge and after discharge until 36 months postoperatively.

Intraoperative categories comprise the duration of the surgical procedures, the expertise of the responsible surgeon, and complications and findings during surgery.

Within the postoperative setting until discharge, the frequency of persistent hyperparathyroidism, the morbidity and the length of hospital stay are noted. During follow up, the frequency of persistent or recurrent sHPT, the frequency of subsequent parathyroid autotransplantation due to refractory hypoparathyroidism, the frequency of reexploration of the neck or the parathyroid autograft, the frequency of irreversible paralysis of the recurrent laryngeal nerve, the change of symptoms related to sHPT as well as the change in quality of life and finally the incidence of death are recorded.

### Trial population

Patients on long-term (>12 months) dialysis with sHPT (PTH ≥ tenfold above normal value) either with hypercalcemia (developing spontaneously or under treatment with Vitamin D) or with normocalcemia in coincidence with renal osteopathy.

### Eligibility

Detailed inclusion and exclusion criteria are specified in table 1.

**Table 1:** Subject Inclusion and Exclusion Criteria

**Inclusion criteria**	**Exclusion criteria**
• Patients on long term dialysis treatment (>12 months) with sHPT	• Primary or Tertiary hyperparathyroidism (hyperparathyroidism after kidney transplantation)
• PTH ≥ tenfold above upper normal value	• Familial hyperparathyroidism(MEN I, MEN II, hereditary hyperparathyroidism)
• Age equal or greater 18 years	• History of neck explorations for thyroid/parathyroid disorders
• Informed consent	• Malignant disease of the thyroid glands
	• Bleeding disorder/coagulopathy
	• Severe psychiatric or neurologic disease
	• Drug- and/or alcohol-abuse
	• Participation in another intervention-trial with interference of intervention and outcome
	• Inability to follow the instructions given by the investigator (e.g. insufficient command of language)

Patients are screened consecutively for eligibility in all participating centres after approval of the study protocol by the local ethics committee. A contract has been signed by the Study Centre of the German Surgical Society in Heidelberg (SDGC) and the participating hospitals for correct conduction of the trial according to Good Clinical Practice. All participating surgeons performing the interventions have been instructed by detailed manuals.

### Consent

Patients who are scheduled for parathyroidectomy because of sHPT will have a pretreatment visit to give informed consent. During this visit the patient will be screened and informed about the TOPAR PILOT trial. In this conversation with the patient the study procedures, risks, benefits and data management will be clarified in detail.

### Procedures for minimizing bias and blinding

Several appropriate methods are used in order to minimize random and systematic sources of bias.

In order to achieve equal groups of patients free from systematic selection bias, an internet-based computer randomization is performed after informed consent at each centre. The main responsibility for the randomization process lies at the SDGC in Heidelberg.

The internet-based computer randomization is performed using the Randomizer for Clinical Trials 1.6.0, developed at the Institute for Medical Informatics, Statistics and Documentation at Medical University of Graz, Austria. The randomization is stratified by centres and is done to generate equal treatment groups by minimizing selection bias. Stratification for the participating clinical sites reduces bias due to centre effects. The trial is only performed in high volume centres (defined as more than 15 parathyroidectomies per year for sHPT) minimizing bias due to learning effects. No additional surgical training has to be done as the neck exploration is the same in TPTX+AT as in the standard procedure and in TPTX.

In order to obtain blinding in this trial a shame procedure would be mandatory in the TPTX group for fake autotransplantation which is in this case unethical due to the design of a non confirmatory study.

### Interventions

#### Preoperative interventions

Patients eligible to participate will be asked to give informed consent. Patients will be randomised into the following treatment groups thereafter.

Consecutively, baseline data will be documented at screening visit, including past medical history, current specific medication and current symptoms of sHPT. A quality of life questionnaire (SF-36) will be handed out preoperatively.

#### Surgical interventions

The responsible surgeon will document any particularities of the surgical intervention and the estimated number of similar operations he/she has performed so far. A standardized surgical approach to the parathyroids will be performed in both groups:

With the patients under endotracheal anesthesia and with the neck hyperextended, a Kocher's incision is made, preferably in a skin crease. The incision line should be drawn preoperatively, two fingerbreadths above the suprasternal notch when the neck of the patient is extended. The subcutaneous layer and platysm muscle is cut with electric cautery. The upper flap is raised to the upper border of the thyroid cartilage. The lower flap is mobilized to the suprasternal notch. A retractor system is used to local standards. The cervical fascia is incised in the midline. The sternothyroid and sternohyoid muscles are retracted or divided. On both sides, thyroid lobes are rotated medially, and the middle thyroid veins are divided. The lobes are retracted medially one after another and the inferior thyroid arteries and recurrent laryngeal nerves are identified.

#### TPTX Group

After mobilization of the thyroid gland, the parathyroid glands have to be identified within the cricothyroid space or in the thyrothymic tongue. Resection of all four (or more) parathyroid glands is performed, carefully avoiding any damage to the parathyroid capsules. The surgeon must change gloves to avoid seeding of parathyroid cells after preserving a part of each gland for pathology. Thymectomy on one side is only performed, if only one gland is found in the respective side or if palpation reveals a suspicious mass within the thymus.

#### TPTX+AT Group

Performance of TPTX, supplemented by bilateral routine cervical thymectomy as complete as possible.

Autotransplantation (AT): a small incision is made over the brachioradial muscle of the non-shunt-bearing forearm (alternative: muscles of the lower limb). After cutting approximately 50 mg of the most normal appearing, e.g. smallest, preferably nonnodular parathyroid gland into pieces of 1 mm^3 ^in size, 20 of these pieces are placed in single muscle pockets each of the brachioradial muscle which are closed by clips thereafter.

#### Histology procedures and crypreservation of resected specimen (both groups)

A small part of each gland has to be sent to pathology for frozen section in order to confirm organ diagnosis. The remaining parathyroid tissue will be placed in cold sterile saline solution for later cryopreservation. Cryopreservation is performed by placing cut pieces of the selected parathyroid gland into a vial containing a solution of 80% culture media, 10% patient serum and 10% dimethyl sulfoxide. The vial is then cooled to 70°C at a rate of 1°C per minute.

#### Postoperative interventions

At day of discharge from hospital, the laboratory parameters and current specific medication are documented again. Patients are asked to fill out another quality of life questionnaire (SF-36). Assessment of vocal cord function has to be documented.

After discharge a follow up visits at 6, 12, 24 and 36 months postoperatively are performed. Follow-up visits consist of a telephone call, a written questionnaire (medical history, current specific medication and specific symptoms and a quality of life questionnaire (SF-36)) to the patient, who is asked to complete it with the help of their general practitioner or nephrologists. The follow up is under the responsibility of the centres under supervision of the SDGC.

**Figure 2:**
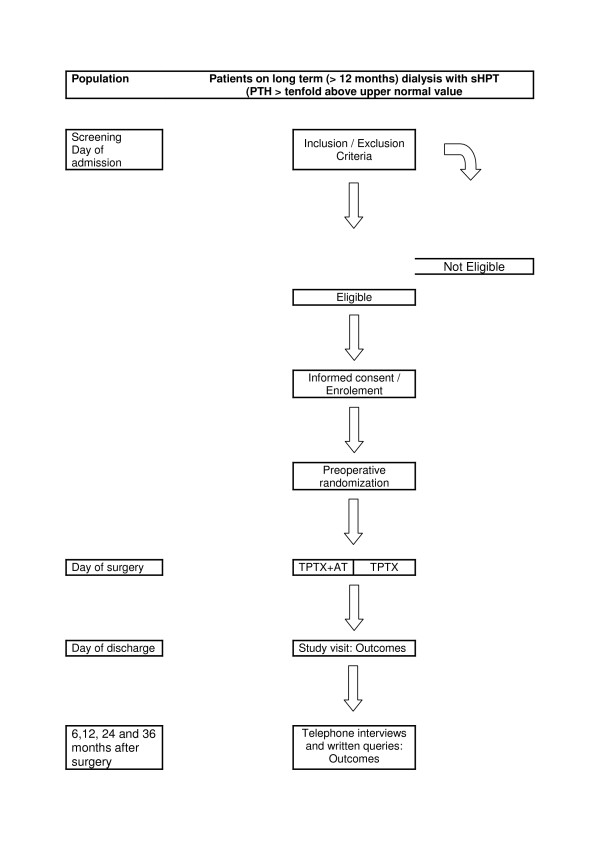
This figure represents a flowchart comprising all the interventions of the TOPAR PILOT trial.

Figure 2 shows a flow-chart of the interventions.

### Number of patients needed

This trial is designed on the basis of a critical appraisal of the existing evidence. The observed difference between the recurrence rates of TPTX+AT and TPTX is of clinical relevance. To assess both methods a multicentred randomized controlled trial is mandatory.

A pilot study design was chosen for the following reasons: In the context of a noninferiority design with a binary primary endpoint using the confidence interval approach for the odds ratio a sample size of 2 × 656 = 1310 patients would be needed to detect non-inferiority with δ = 1588 based on equal risks of recurrence in both groups (or lower in TPTX) with a power of 80% and a significance level of 5% (Monte-Carlo simulation using logistic regression). Incorporating a rate of drop-outs of 15%, 2 × 770 = 1540 patients would be needed. In case of slight differences between the centres the power will not change significantly. Assuming that a risk of 12,5% in the TPTX+AT group is not inferior based on the risk rate of 10% in the TPTX group (difference of 0.025 or an odds ratio of 1.286) would lead to a sample size of more than 4000 and is therefore far from feasibility. The latter margin is the same as usually used for bioequivalence trials (change of 80% or 125%). Therefore better estimates are needed and a pilot multicentred randomized controlled trial comparing 50 versus 50 randomized patients seems feasible and ethical to assess the recurrence rate of both methods after three years in specialized centres.

### Clinical sites

Sites are selected according to their experience in parathyroid surgery and willingness to adhere to the clinical trial protocol. The surgical expertise of each participating surgeon is documented and taken into account during the analysis of the trial data.

### Safety aspects

A small part of each gland is sent to pathology for frozen section in order to confirm organ diagnosis. The success of the intervention is assessed by perioperative monitoring of PTH and calcium recorded in the CRF. All participating centres guarantee cryopreservation of parathyroid tissue from each patient to enable possible subsequent parathyroid autotransplantation, if necessary. If postoperative persistent hypoparathyroidism refractory to medical treatment occurs, reimplantation is performed. The necessity of reimplantation is discussed and defined in each individual case by a medical clinical expert board. Therefore, clinical symptoms (e.g. repeated episodes of paraesthesia and seizures or others), the necessity and amount of medication to achieve normocalcemia and laboratory data (PTH, calcium) will be taken into consideration.

All serious adverse events (SAE) have to be reported to the principal investigator and the leading ethic committee. For the safety analysis the incidence of adverse events (AE) and SAE are analyzed. Patients may be withdrawn from the study at any time either at their own request or at the request of the principal investigator.

Safety evaluation will be done. The adverse events of both treatment groups will be demonstrated in comparison considering severity and causality. Safety interim analyses are planned each year and the independent data and safety board will follow the progress of the trial to control the safety.

### Statistical Analysis

Statistical methods are used to assess the quality of data, homogeneity of treatment groups, endpoints and safety of the TPTX versus TPTX+AT technique. The analysis is performed on the basis of an intention to treat (ITT) population and with respect to ITT principles. A patient belongs to the ITT population, when the patient was found to be suitable for the study, signed the informed consent form and was randomized thereafter.

#### Descriptive analysis

Baseline characteristics as well as efficacy and safety endpoints will be analyzed with descriptive methods. The description of continuous variables includes at least: number of observations, mean, standard deviation, median, minimum and maximum in the trial population. The description of nominal variables includes at least the number and percentage of patients belonging to the relevant categories in the trial population as well as in each treatment group. In order to improve the presentation of the observed data, graphical methods will be applied.

### Trial organization, quality control and registration

TOPAR PILOT was designed at the Department for Visceral, Thoracic and Vascular Surgery, Philipps-University, Marburg in cooperation with the Study Centre of the German Surgical Society (SDGC), Heidelberg and the Institute of Medical Biometrics and Informatics (IMBI) at the University of Heidelberg.

Quality assurance will be done in cooperation with the Network of Coordinating Centres for Clinical Trials (KKS).

The trial is monitored by an independent monitor of the KKS in Heidelberg. The trial is performed according to the Declaration of Helsinki in its current German version and the Good Clinical Practice (GCP). Before the start of the trial the independent ethics committee of the Philipps-University, Marburg gave a positive vote on August 21th 2006. The trial was registered on August 22d in 2006 at the International Standard Randomized Controlled Trial Number Registration (ISRCTN86202793).

### Financial support

This trial is funded by the German Federal Ministry of Education and Research [Bundesministerium für Bildung und Forschung (BMBF); 01GH99033] and the German Surgical Society [Deutsche Gesellschaft für Chirurgie (DGCH)]. Additional industrial funding for the infrastructure of the SDGC is received from Ethicon GmbH, Norderstedt, Germany, and Tyco Healthcare Deutschland GmbH, Neustadt, Germany.

### Current status and duration of the trial

Up to now, 2 centres are initiated. The first patient was randomized on January 24th 2007. The expected recruitment period will last 18 months.

## Discussion

TOPAR PILOT is a randomized controlled multicentred pilot trial to generate high quality evidence comparing the outcomes of TPTX+AT vs. TPTX. Since secondary hyperparathyroidism is a very common disease in patients with chronic kidney failure, even a small difference in outcomes will be of clinical relevance. If TPTX proves to have no relevant disadvantages and a smaller rate of recurrent sHPT, a large scale confirmatory study will be launched to establish this approach.

Recent reports comparing TPTX+AT to TPTX were retrospective, underpowered or not designed as RCT's. No unique definition for recurrent sHPT was used to calculate the rate of recurrent sHPT. The follow up periods of former studies have often been too short and were not standardized (table 2). Therefore we will perform a follow up of each patient at 6, 12, 24 and 36 months after surgery to allow an assessment of the medium/long term effect of the different surgical treatments.

**Table 2:** Rate of recurrent sHPT after TPTX or TPTX+AT

**Reference**	**TPTX+ AT n**	**TPTX n**	**Routinely performed thymectomy**	**Mean length of follow – up (months)**	**Recurrent sHPT^1 ^(%)**
Rothmund et al. 1991 [29]	20		yes	43	0
Zaraca et al. 1999 [58]	18		no, only if palpation was suspicious or parathyroid gland was missing	28	10
Walgenbach et al. 1997 [46]	67		yes	18	4.5
Walgenbach et al. 1998 [59]	86		yes	24	3.5
Henry et al.1990 [40]	152		no	30	10.5
Welk and Alix 1987 [60]	21		n.m.	33	24
Hampl et al. 1991 [37]	13		yes	33.5	76.5
Chou et al. 2002 [61]	75		n.m.	54	13.3
Bessell et al. 1993 84	42		n.m.	40	9.5
Dotzenrath et al. 2003 [62]	99		yes	51	6
Korzets et al. 1987 [63]	19		n.m.	6–60	26.3
Kinnaert et al. 2000 [31]	59		no	38	12
Tominaga et al. 1997 [39]	519		yes	36	10
				60	20
Ockert et al. 2002 [32]	11		no	38	45
		11	no	22.5	0
Kaye et al. 1993 [64]		13	n.m.	46	0
Higgins et al. 1991 [44]	34		no	72	80
		9	no	10	0
Nicholson 1996 [36]	13		no	24	16
		24	no	24	0
Saunders et al. 2005 [52]		55	no	29	4
Stracke et al. 1999 [35]		20	n.m.	20	4
Hampl et al. 1999 [65]		11	no	26	0
Ljutic D et al. 1994 [66]		43	no	104	2.3

n.m. = not mentioned

^1 ^Recurrent sHPT is defined as an increase of PTH > 5 fold above normal value after more than 6 months after initial PTX and after initial (postoperative) normalization of PTH.

The evaluation of optimal surgical options and procedures should be performed according to the best current knowledge. Due to the principles of Evidence-based Medicine (EbM), the randomized controlled trial is assumed to be the "Gold Standard" in evaluating different medical methods and procedures. Even in RCT's, the risk of bias should not be underestimated. In surgical trials a double blinded trial design is difficult to establish. In this study, even a patient-blinded trial design would necessitate a shame procedure at the forearm for fake autotransplantation in the TPTX-group which is in this case unethical due to the design of a non confirmatory study.

However, the patient selection, power, randomization, surgeon's skill, data assessment and documentation of outcome measures and statistical analysis are important design issues and susceptible to the different forms of bias. Hence, all these issues are specified in the trial protocol a priori.

### Selection bias

To minimize selection bias all patients scheduled for parathyroidectomy because of sHPT are screened consecutively in all participation sites. Moreover, neither patient age nor the amount of PTH-secretion is an exclusion criterion. Screening lists and case report files are monitored by independent investigators from the SDGC to ensure correct patient recruitment and meticulous documentation.

### Randomization bias

Randomization is done using in an internet-based computer randomization. The randomization will be stratified by centres and will be done to generate equal treatment groups by minimizing selection bias. Stratification for the participating clinical sites will reduce bias due to centre effects.

### Surgeon's expertise bias

In this study, stratification for surgeon's skill is made and measured data will be analyzed by analysis of variance in regard to surgeon's skill and centre. Due to this analysis of variance, bias of centre effects and surgeon's skill should be reduced.

The trial will only be performed in high volume centres (defined as more than 15 parathyroidectomies per year for sHPT) minimizing bias due to learning effects. No additional surgical training has to be done as the neck exploration is the same in TPTX as in the standard procedure of TPTX+AT.

In conclusion, this is the first randomized controlled multicentred trial comparing different surgical techniques in parathyroid surgery for sHPT. If significant, the results will be revised by a large scale confirmatory study. The results may change the current surgical practice thereafter.

## Abbreviations

CAEK - Chirurgische Arbeitsgemeinschaft Endokrinologie (Study Group of Endocrine Surgeons);

KKS - Koordinierungszentrum für Klinische Studien (Coordination Centre for Clinical Trials);

MRCT - multi-centre randomized controlled trial;

PTX - Parathyroidectomy;

SDGC - Studienzentrum der Deutschen Gesellschaft für Chirurgie (Study Centre of the German Surgical Society);

sHPT - secondary hyperparathyroidism;

SPTX - subtotal parathyroidectomy;

TPTX+AT - Total parathyroidectomy with autotransplantation and with routinely performed thymectomy;

TPTX - Total parathyroidectomy without autotransplantation and without routinely performed thymectomy;

PTH - Intact parathyroid hormone.

## Competing interests

The author(s) declare that they have no competing interests.

## Authors' contributions

KS conceived of the study, made substantial contributions to conception and design as well as to the acquisition and interpretation of data. Moreover, she has been essentially involved in drafting the manuscript.

JAV participated in the sequence alignment and partly drafted the manuscript.

SW participated in the design of the study and performed the statistical analysis.

ED participated in the sequence alignment and helped to draft the manuscript.

NV participated in the design of the study and was mainly involved in the analysis and interpretation of data as well as in the performance of the statistical analysis.

HPK participated in the design of the study and its coordination and helped to draft the manuscript.

CMS made substantial contributions to conception and design, has been involved in drafting the manuscript and revised it critically for important intellectual content.

MR conceived of the study, has been involved in drafting and revising the manuscript and gave final approval of the version to be published.

All authors read and approved the final manuscript.
